# CD8-Lymphocytic Phenotype Significance in Primary Multiple and Familial Melanoma with Various CDKN2A Mutational Status

**DOI:** 10.3390/medicina59122151

**Published:** 2023-12-12

**Authors:** Luana-Andreea Boşoteanu, Emma Gheorghe, Mariana Aşchie, Georgeta Camelia Cozaru, Mariana Deacu, Gabriela Izabela Bălțătescu, Cristian Ionuț Orășanu, Mǎdǎlina Boşoteanu

**Affiliations:** 1 Department of Dermatovenerology, “Elias” Emergency University Hospital, 011461 Bucharest, Romania; 2 Institute of Doctoral Studies, Doctoral School of Medicine, “Ovidius” University of Constanţa, 900573 Constanţa, Romania; 3 Department of Dermatology, “Sf. Apostol Andrei” Emergency County Hospital, 900591 Constanţa, Romania; 4 Department of Histology, Faculty of Medicine, “Ovidius” University of Constanţa, 900527 Constanţa, Romania; 5 Clinical Service of Pathology, “Sf. Apostol Andrei” Emergency County Hospital, 900591 Constanţa, Romania; 6 Department of Pathology, Faculty of Medicine, “Ovidius” University of Constanţa, 900527 Constanţa, Romania; 7 Department VIII—Medical Sciences, Academy of Romanian Scientists, 030167 Bucharest, Romania; 8 Center for Research and Development of The Morphological and Genetic Studies of Malignant Pathology (CEDMOG), 900591 Constanţa, Romania

**Keywords:** familial melanoma, multiple primary melanoma, CD8, CDKN2A mutation, immunohistochemistry

## Abstract

*Background and Objectives*: In the realm of the rising incidence of cutaneous and mucous melanoma, CDKN2A mutations characterize familial and multiple primary melanoma cases. The involvement of tumor-infiltrating lymphocytes (TILs) is interconnected with survival rates, but may extend even further. The aim of this study is to verify the accuracy of the classical “naked eye” count of CD8-positive T cells comprised within the tumoral population and peritumoral infiltrate versus that obtained via a special software run by the aid of artificial intelligence (AI), used to determine the percentage of CD8-positive TILs. *Materials and Methods*: The present retrospective cross-sectional study conducted over a period of 5 years (2018–2022) focused on patients diagnosed with mucous and/or cutaneous melanoma, with a positive family history for melanoma, or personal antecedents of primary malignant melanocytic lesions. The 23 selected cases were diagnosed histopathologically, tested for CDKN2A mutations through fluorescent hybridization in situ, and CD8 immunohistochemistry was performed. The included slides were evaluated both manually (naked-eye examination) and automatically (via QuPath platform) for quantifying the CD8-positive TILs. *Results*: The number of CD8-positive TILs in melanoma samples has been more accurately identified through the use of an AI-mediated software as compared to the human-eye evaluation performed by experimental pathologists. A higher percentage of CD8-positive intratumoral lymphocytes versus stromal lymphocytes was positively associated with more numerous metastatic sites. *Conclusions*: The CD8 lymphocytic phenotype harbors major significance in the context of familial and multiple primary melanoma and may comprise a cost-effective investigation meant to help in the establishment of melanoma prognosis and response to immunotherapy.

## 1. Introduction

Melanoma is considered to be an aggressive malignant tumor, encapsulating the frequent loss of differentiation markers, associated with accelerated evolution and limited survival rates. The available data regarding its incidence highlight a twofold increase every decade [1]. Familial melanoma represents a relatively commonly encountered entity in the global nosological context of melanoma, being situated in direct relationship with cyclin-dependent kinase inhibitor 2A (CDKN2A) and cyclin-dependent kinase 4 (CDK4), two genes essentially involved in cellular division, whose potential alterations increase the risk of occurrence of melanocytic malignancies [2]. Inside the diverse tumor microenvironment, T cells account for a significant part of the immune infiltrate, being composed of effector, memory, and regulatory T cells [3]. There is also a group of CD8 T cells that harbor dysfunction, characterized by a deficit of the standard effector capacities (e.g., cytotoxicity) and by a particular cytokine secretion model [4]. Furthermore, the state of exhaustion is commonly observed; even though it has been formerly thought to be correlated with a loss of proliferative capacity, T cells exhibiting high levels of PD-1 expression have been shown to encompass superior proliferative abilities [5].

CD8-positive T cells are frequently observed in T-cell lymphomas, some of them post-thymic [6], nodular lymphocyte predominant Hodgkin lymphoma [7], heterotopic ovarian splenoma, melanoma, mycosis fungoides, splenic hamartoma, and scarcely in B-cell chronic lymphocytic leukemia, lymphomatoid papulosis [8], and mantle cell lymphoma [9,10]. Given the fact that Galon and Bruni reviewed that the fidelity of the standard TNM system may be surpassed by the type, density, and localization of immunocompetent cells at the tumoral level in colorectal cancer, a major goal of the present study is represented by the analysis of the immune particularities of T cells in melanoma [11]. Another factor that has been recently recognized as positive predictive marker for response to immune checkpoint therapies is represented by the tumor mutation burden, which has been proven to also engage in immune checkpoint blockade inhibition [12].

The four main histopathological subtypes of melanoma comprise: superficial spreading melanoma (SSM) and nodular melanoma (NM), which represent the two most common melanoma subtypes [13], followed by acral lentiginous melanoma (ALM), frequent in the African American population, and lentigo maligna melanoma, which generally affects sun-exposed areas of the skin [14]. In general, a higher level of CD8 positivity is associated with better outcomes in SSMs [15], while NMs overexpress genes that initiate an immune response to tumor antigens [16]. CD8 positivity can still be present, but the overall immune response is linked to more favorable responses to anti-PD1 agents in NMs [17]. An analysis of individuals diagnosed with ALM indicated that positive survival outcomes demonstrated the tendency to be linked with elevated levels of TILs [18].

Moreover, the aim of this study is to verify the accuracy of the classical “naked eye” count of CD8-positive T cells comprised within the tumoral population and peritumoral infiltrate versus that obtained via a special software run by the aid of artificial intelligence, used to determine the percentage of CD8-positive TILs.

## 2. Materials and Methods

We conducted a retrospective cross-sectional study, over a period of 5 years (2018–2022), on patients diagnosed with mucous and/or cutaneous melanoma, with a positive family history for melanoma, or personal antecedents of primary malignant melanocytic lesions. Thus, we scanned the physical records within the archive and the digital database from the Clinical Service of Pathology, Emergency Clinical County Hospital of Constanţa, located in South-Eastern Romania.

The inclusion criteria comprised adult patients (over the age of 18 at diagnosis) with mucous and/or cutaneous melanoma confirmed histopathologically in the context of familial melanoma and/or multiple primary melanomas. The exclusion criteria referred to in situ melanomas, underage patients, those with melanoma detected in visceral sites, without other affected family members, or without previously diagnosed primary melanomas.

Afterwards, epidemiological and clinical data, such as sex, age at diagnosis, area of residence, anatomical site of melanoma development, number of pigmentary lesions, and elected treatment were extracted from the medical files. This information was evaluated by the attending dermatologist and the required excisions were performed by the plastic surgeon.

The tissue samples obtained after the elliptical surgical excision and re-excision of the scars were described from a macroscopical point of view and processed according to standard protocols, in order to acquire hematoxylin and eosin (H&E)-stained slides. The latter were evaluated by experienced pathologists within the Clinical Service of Pathology and essential characteristics (histopathological melanoma subtype, Breslow index, mitotic rate, presence/absence of lymphovascular and perineural invasion, type of lymphocytic infiltrate) were noted.

The most representative slides were selected for immunohistochemistry, that was performed on 23 cases. The 4 μm sections derived from formalin-fixed paraffin-embedded specimens underwent successive phases of deparaffinization in xylene and rehydration in alcohol solutions of progressive descending concentrations. The heat-induced epitope retrieval method was employed for epitope discovery. The slides were incubated with the CD8 marker (Clone SP16, Master Diagnostica, Sevilla, Spain). Marking was performed using chromogen 3,3′-diaminobenzidine, whereas counterstaining was obtained with hematoxylin. Finally, the slides were dehydrated and clarified before being mounted. Regarding the evaluation of tumor-infiltrating lymphocytes (TILs), a specific region of focus was chosen among the tumor invasion area in each sample and scored as 0 or 1, according to the absence or presence of CD8-positive cells, respectively. Two experimental pathologists, who were not aware of the outcomes and therapeutic response of each patient, manually examined the tissue samples, focusing on TILs. Lymphocytes in direct contact with the tumor margins or comprised within the malignant cellular population were labelled as ‘intratumoral’, while those situated externally were considered ‘peritumoral’.

All slides were scanned using the Huron LE120TM 4000XT scanner (Huron Technologies International Inc., St. Jacobs, ON, Canada) at the Center for Research and Development of the Morphological and Genetic Studies of Malignant Pathology (CEDMOG). For each slide, a whole slide image (WSI) was obtained and, afterwards, visualized by the HuronViewer™ Software, while the image analysis was performed using the QuPath Software platform (version 0.4.3). The QuPath method for WSIs includes the following steps: image import, slide navigation, annotation of regions of interest, automated cell detection within annotated regions, and quantitative analysis of positive cells. Therefore, QuPath uses proprietary artificial intelligence, based on in-built machine learning for image analysis. The AI-generated report was used for comparison with the results obtained with the aid of the human eye. The results were reported in percentages of positive nuclei relative to 100 examined lymphocytic nuclei, at 400× magnification. The reactions were considered positive when >5% of TILs harbored different degrees of staining intensity. Aiming to maintain the accuracy of the method and further results, positive control slides were provided for every determination.

The technique for molecular biology testing of the CDKN2A status was implemented in CEDMOG, using dual-color fluorescence in situ hybridization (FISH). The formalin-fixed paraffin-embedded samples of the 23 selected cases were processed with the aid of the chromosome 9 centromere (Table 1) in order to detect the percentage values for p16/CDKN2A deletion.

The entire informational panel derived from the present research was summarized and standardized in a Microsoft Excel sheet (Microsoft, Redmond, WA, USA). The same program was employed to process the data and to calculate the *p*-value for the appropriate associations. We also used SPSS Statistics version 26 (IBM Corporation, New York, NY, USA) for reliability analysis and intraclass correlation coefficient estimation; a *p*-value inferior to 0.05 was considered statistically significant.

Confidentiality of the patients was respected during the deployment of the present research, in compliance with the regulations stated by the Declaration of Helsinki. All subjects gave their informed consent for inclusion before their hospitalization at our institution, and the protocol was approved by the Ethics Committee of “Ovidius” University of Constanţa.

## 3. Results

**Figure 1:**
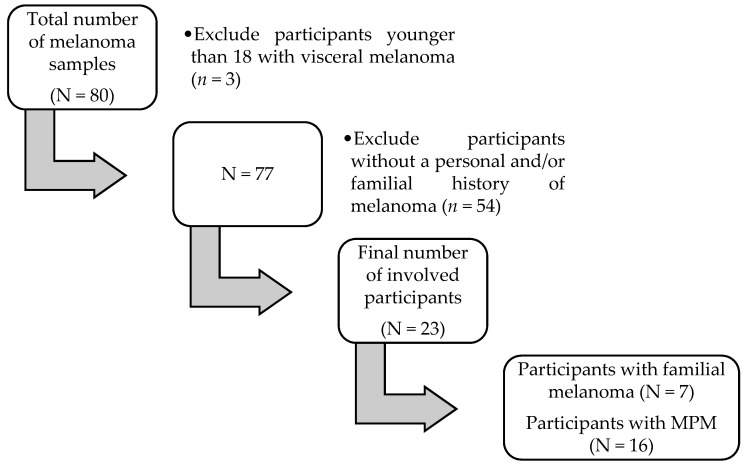
Flowchart of data selection.

After the preliminary search among medical records, we identified 80 patients with a histopathological diagnosis of melanoma. After the application of inclusion criteria, we excluded 3 patients with visceral melanoma and 54 others that did not exhibit either personal or familial characteristics superposable to CDKN2A positivity; no case of melanoma in situ was detected in the analyzed individuals. Hence, the final group over the period of 5 years comprised 23 treatment-naïve subjects—7 individuals emerging from families with at least one other first-degree relative diagnosed with melanoma, and 16 patients with multiple primary melanoma (MPM) (Figure 1).

### 3.1. Demographic, Clinical and Histopathological Characteristics

The mean age at diagnosis was 62.30 ± 14.77 years old, with an almost equal gender distribution (male:female ratio 1.09:1). A prevalence of cases was encountered in the eighth (30.43%, *n* = 7) and seventh (21.74%, *n* = 5) decade of life, and more than half of the patients resided in urban areas (60.87%, *n* = 14). The most common anatomic site was the anterior/posterior thorax (39.13%, *n* = 9), followed closely by the involvement of the head and neck cutaneous areas (34.78%, *n* = 8). The demographic and clinical particularities of the studied patients are depicted in Table 2.

Regarding the histopathological aspects of the selected melanoma fragments (Table 3), the majority of cases corresponded to superficial spreading melanomas (56.52%, *n* = 13), while nodular melanomas were diagnosed in nine cases (39.13%). The mean Breslow depth measured 4.88 ± 3.92 mm, a value derived from the preponderance of tumoral populations surpassing 4.01 mm on vertical sections (47.82%, *n* = 11). Ulceration was predominant in the analyzed cases and equally as frequent as lymphovascular invasion (56.52%, *n* = 13), while perineural invasion and regression were only encountered in 21.73% (*n* = 5) and 17.39% (*n* = 4) of cases, respectively. The average mitotic rate determined in the particular melanoma samples was 3.04 ± 3.55 per mm^2^, most of the cases harboring between 1.00–1.99 mitoses/mm^2^ (34.78%, *n* = 8) and ≥5.00 (34.78%, *n* = 8).

### 3.2. Immunohistochemical Findings of CD8 Results in the Peritumoral Infiltrate

The staining pattern of CD8-prelucrated specimens was evaluated manually by two experimental pathologists, who were not aware of the outcomes and therapeutic response of each patient. Afterwards, QuPath was used to scan the same slides, in order to obtain an AI-generated report, used for comparison with the results obtained with the aid of the human eye. Concerning the manual evaluation, the average percentage of peritumoral CD8-positive nuclei in the analyzed cases was 13.47% ± 22.81, whereas the average percentage of intratumoral nuclei displaying CD8 immunohistochemical positivity was 20.86% ± 24.65.

In the examined specimens, 13 cases of SSM, 9 NMs, and 1 ALM were diagnosed. The average percentage of intratumoral and peritumoral CD8-positive TILs in the SSM category was 14.23% and 16.53%, respectively. On the other hand, NMs presented an average percentual value of 26.11% regarding the intratumoral TILs with CD8 positivity and 10.55% CD8-positive T cells in the peritumoral infiltrate. The singular case of ALM revealed 60% CD8-positive intratumoral TILs and no CD8 positivity in the peritumoral cellular environment. 

**Figure 2:**
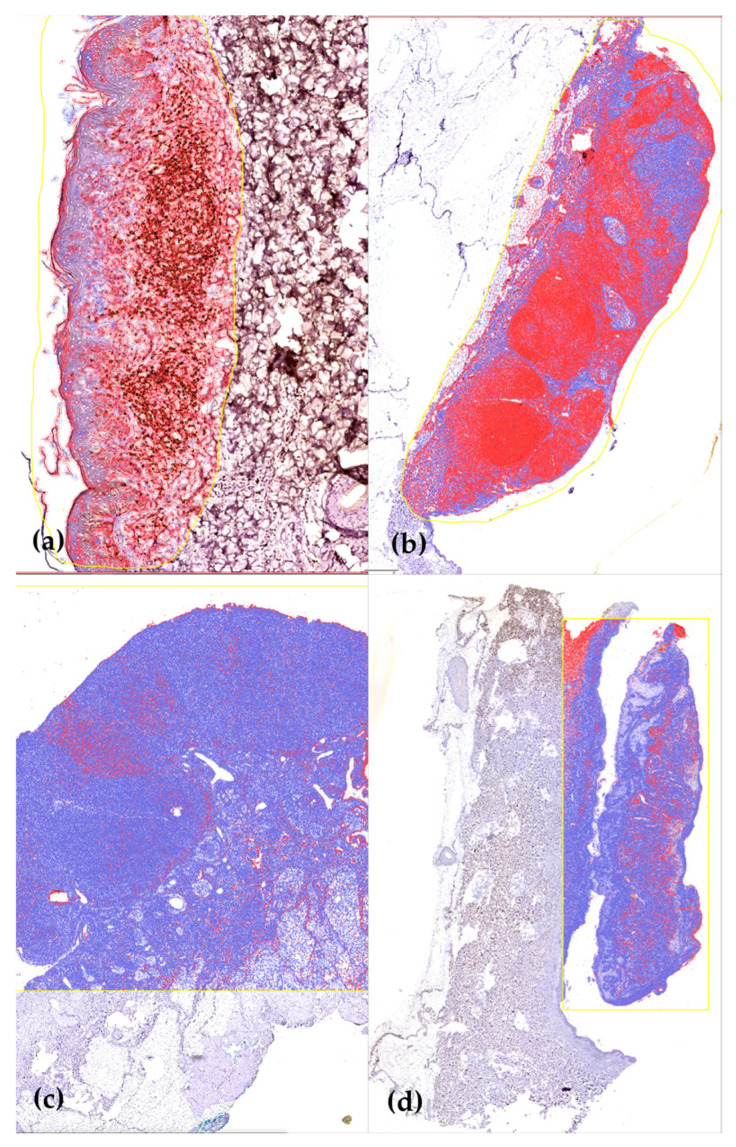
Automatic specimen evaluation (via QuPath): (**a**) Highest CD8 positivity encountered in 96.24% of lymphocytes; (**b**) CD8 positivity recorded in 64.84% of the peritumoral cellular infiltrate; (**c**) Lowest percentage of CD8-positive lymphocytes (7.85%); (**d**) CD8 positivity in 12.47% of the peritumoral population.

Out of the 23 CD8-stained cases, only 13 (56.52%) could be supplementarily examined via an AI-mediated platform in an accurate manner (Figure 2); the other 10 cases did not generate quantifiable results, due to tissue artifacts generated in the preanalytical phase or by the analytical processing. The automated analysis of the selected fragments generated an average percentage of peritumoral CD8-positive nuclei of 45.49% ± 25.07, while the results of naked-eye evaluations provided an average of 24.23% ± 27.23.

### 3.3. Differences between the Immunohistochemical Expression of CD8 in the Peritumoral Infiltrate Assessed by a Human Examiner Versus an Artificial Intelligence Platform

The inter-item covariance matrix (Table 4) reveals a positive linear relationship between “QuPath” and “Human”, as the covariance (99,236) is substantial, indicating that when one variable tends to deviate from its mean, the other also tends to do so. Additionally, both “QuPath” and “Human” exhibit notable variance, signifying considerable variation within each variable. These findings suggest a meaningful connection between the two items and highlight their individual variability.

The **intraclass correlation coefficient** (ICC) for **single measures** is approximately 0.134, with a 95% confidence interval ranging from −0.429 to 0.622. The F Test with a true value of 0 yielded a value of 1.309. These results suggest that there is some degree of correlation among the evaluations performed within the same method. However, the confidence interval spans both positive and negative values, indicating a certain level of variability in the correlation estimate.

For **average measures**, the ICC is approximately 0.236, with a 95% confidence interval ranging from −1.504 to 0.767. The F Test with a true value of 0 also yielded a value of 1.309. These results similarly suggest a moderate level of correlation within the same examination technique, with a somewhat wider confidence interval. The variability in the confidence interval indicates potential fluctuations in the ICC estimate.

Both **single measures** and **average measures** (Table 5) have *p*-values of 0.324, indicating that the ICC estimates are not statistically different from 0 at the 0.05 significance level. Therefore, the data do not provide strong evidence for a significant correlation beyond what would be expected due to random chance. Therefore, we can conclude that the QuPath-mediated analysis of TILs provides better and higher values than the human-eye examination, but the differences are not statistically significant.

## 4. Discussion

To our knowledge, the present research is the first of its type to approach the clinical, evolutionary, and immunophenotypic correlations between the CD8 immunoreaction, familial melanoma, and spontaneous multiple primary melanoma in a cohort of Romanian patients. This analysis highlighted the value of CD8-positive TILs as being more accurately revealed through automatic AI-aided examination, compared to the human-eye evaluation (with an average of 45.49% of positive nuclei detected vs. 24.23%), despite the absence of statistically significant results regarding the quantitative evaluation of CD8-positive TILs. In further studies, the number of examined cases will be extended, therefore aiming to verify changes in statistical significance.

The significant relationship between CDKN2A expression and tumor immunity has been postulated by Chen et al., given the association of TIL levels with the molecular expression of CDKN2A [19]. Therefore, the potential molecular role of this genetic mutation over the survival rates registered in CDKN2A-positive patients should be further investigated in order to confirm possible action mechanisms that could be targeted by novel specific therapies.

A study by Yuan et al. showed that the high degree of CD8-positive T cells in the tumoral or peritumoral infiltrate is correlated with a better prognosis due to the favorable response to therapy [20]. The supposition according to which only certain types of TILs harbor a prognostic value in melanoma patients was supported by the fact that CD8-positive T cells play an essential role in tumor suppression, not only in the context of melanoma, but also in other malignant conditions, such as triple-negative breast cancer [21], advanced gastric cancer [22], and stage II-III colorectal cancer [23]. In our study, there was no statistically significant inverse correlation between the high number of CD8-positive TILs and melanoma progression, this idea being reinforced by cases with multiple visceral metastases and a high percentage of CD8 positivity in the peritumoral infiltrate, as well as by samples from patients without organ metastases that harbored few CD8-positive lymphocytes.

The outcomes of a recent research study indicated that patients exhibiting elevated CD8-positive TILs had an extended overall survival and progression-free survival, suggesting that CD8-positive TILs serve as positive prognostic factors for patients treated with immune checkpoint inhibitors [24]. Moreover, the presence of CD8-positive T cells at the invasive margin showed a negative correlation with the depth of invasion and vascular invasion [25]; following stimulatory immunotherapy, CD8-positive TILs at the invasive margin exhibited greater levels of anti-tumoral activity when contrasted with intratumoral CD8-positive infiltration [24]. Furthermore, clinicopathologic correlations revealed that the presence of infiltrates in melanoma is linked to a more favorable prognosis, when compared to histologically similar tumors lacking such infiltrates [26]. The observations generated by Piras et al., stating that there was a significant contrast in 5-year survival rates among patients with high, moderate, and low CD8-lymphocyte density, irrespective of other clinical variables, suggested that this parameter can be regarded as a crucial and independent prognostic factor [26].

In the study conducted by Zhu et al., CD8 expression was proportional with the detection of CDKN2A mutations among in situ and invasive melanoma samples [27]. Thus, the supposition according to which CDKN2A deletions inhibit the development and proliferation of T-cell infiltration caused by the inhibition of chemokine expression, depending on each phase of the cell cycle, was reinforced. Furthermore, parallel genetic and cellular patterns have been identified in the subgroups exhibiting CDKN2A mutations and low CD8 intensity; the molecular similarity was also conserved in the population without CDKN2A alterations and those cases with noteworthy T-cell expression [17]. These uniform ways of presentation may be explained by the ability of CDKN2A to attract T-cell recruitment and increase the expression of chemokines, supported by mitogen-activated protein kinases (MAPKs) and the nuclear factor kappa-light-chain-enhancer of activated B cells (NF-κB) [17]. In our study, the previously described correlations have also been identified. On average, 5.55% of CD8-positive TILs were detected in fragments with CDKN2A homo- or heterozygous deletions (*n* = 10), while the average percentage of CD8 positivity in the peritumoral infiltrate of cases lacking CDKN2A deletion alterations (*n* = 13) was greater, with a value of 19.61%.

The findings of the Italian Melanoma Intergroup revealed that the density and distribution of CD8-positive cells within the melanoma sample may encompass the future responsivity to MAPK inhibitors (Cobimetinib, Trametinib, Binimetinib), the corollary being that specimens with a low expression of T-cell immune markers correspond to patients with poorer objective response rates, and therefore, with shorter overall survival [28]. This variation in therapeutic resistance/sensitivity may be due to particularities of the immune microenvironment. Its hostile characteristics are thought to originate in the accumulation of macrophages, which might be neutralized with the aid of NF-κB inhibitors [18].

Differences in the response to therapy, encountered in the cohort studied by Li et al. [3], were not only between treatment-naïve and treatment-experimented cases or at the same organ site among the patients not exposed to immune checkpoint blockade therapy [19], but also due to a particular category of ‘dysfunctional’ T cells. Hence, this discrepancy was considered mainly intrinsic, comprised in the tumoral immune environment, probably determined by driver oncogenes and/or the tumor mutational burden [19]. Aside from the first category of T cells, there are other lymphocytes that suffer from “exhaustion”, after repeated cycles of stimulation, consequently restricting their ability to fight infections or to limit the expansion of tumors [29]. The involved pathogenic factors are not completely elucidated; the supposition is that the high energy outputs necessary for the activation of lymphocytes are placed in a competitive state with the prohibitive demands of the tumor growth, which result in the consumption of glucose levels and energy production from mitochondria [30]. A multicentric group discovered that nicotinamide in vitro addition may prevent T cells from entering the exhaustion state, given its properties of energy supply, and even reverse certain modifications that have already taken place [20].

The validation of the immunoscore in melanoma resulted in the following classification: “cold” tumors are defined as non-inflamed, “altered” lesions are considered those with inflammation, but non-infiltrated, while “hot” tumors are typically T-cell-infiltrated proliferations [31]. The former category especially is of interest and may be caused by the absence of expression of the tumor antigen or antigen presentation, deficient retrieval of antigen-presenting cells, or defective activation or trafficking of T cells, which lose their ability to infiltrate the tumor mass [32]. Moreover, the effects of cancer-associated fibroblasts (CAFs) reside in decreasing the permeability of the tumor microenvironment to CD8-positive T cells [33]. A paramount discovery encountered in the study by Han et al. consisted of the association between 9p21 loss and the primary resistance to immune checkpoint blockade therapy (ICT) translated in reduced clinical responsivity [34]. In contrast to 9p21-wild type melanomas, those cases with a loss of 9p21 expression respond 2.8-fold poorer to the administration of ICT [25]. Desfrançois et al. discovered that T cells which have a double-positive CD4 and CD8 profile are identified in almost 60% of melanomas and encompass an original cytokine repertoire, characterized by significant secretion of interleukins 4, 5, and 13 [35]. They are also responsible for the production of tumor-reactive TNF-alpha, due to the recognition of autologous melanoma cells as self-antigens [26]. Therefore, CD4–CD8-positive TILs have been proven as important markers to be targeted by future melanoma therapies. In an analysis conducted by Tumeh and his work group, it is shown that CD8-positive TILs encountered in the intratumoral population of metastatic melanoma samples were associated with high rates of response to PD-1/PD-L1 treatment, documented by radiographic reduction in secondary tumors [36]. Furthermore, those T cells displaying CD8 positivity located at the invasive tumor margin were correlated with the expression of the PD-1/PD-L1 immune inhibitory axis, thus predicting the response to immunotherapy [27].

The differences in CD8 T cell counts between human examination and AI-mediated evaluation may be due to the variations in colorimetry, depth perception, understanding and interpretation of the presented images, figure complexity, and quality. On the other hand, the high variability detected among multiple clinical trials may be explained by the frequent use of small samples (1 mm diameter); its correction might be easily achieved through the examination of biopsies measuring more than the aforementioned dimensions, that become depictive of the degree of CD8 T-cell infiltration [37]. However, due to the stable results and reproducibility of the computerized analyzer of whole-slide images (WSIs), it tends to gain more confidence, compared to the persistent inter-examiner variability encountered in standard visual pathological evaluations [38].

Following the preliminary evidence applicable to breast cancer stating that intratumoral CD8-positive T cells are linked to the negative influence over disease-free survival rather than stromal CD8-positive T cells, given the connection with the overexpression of PD-1/PD-L1 pathway [39], we highlighted that, in melanoma, peritumoral CD8-positive T cells respect the same pattern. An average percentage of CD8-positive intratumoral lymphocytes of 23.33%, as opposed to a mean value of 4.61% positive CD8 stromal cells, was correlated—in our analysis—with the presence of non-regional lymph node metastases and secondary determinations in other organs, most frequently the lungs, liver, brain, and peritoneum. Therefore, these discoveries confirmed the superior characteristic of intratumoral CD8-positive lymphocytes as a negative prognostic marker in comparison with the weak and inconsistent association between peritumoral CD8 positivity and unfavorable evolutionary patterns. However, the prognosis is highly dependent on the stage at diagnosis and various histopathological factors (such as the Breslow index, presence or absence of vascular/perineural invasion) and should also be adjusted for these variables, especially due to the fact that the follow-up interval of time (2018–2022) taken into account in the present study was not sufficiently long in order to thoroughly assess the evolution of each case. It was demonstrated that patients with optimal responsivity to anti-PD1 agents present a significant fraction of memory/resident memory-scoring TILs, which continued to increase after treatment, whereas non-responsive individuals displayed a conflicting phenomenon characterized by early T cell activation, without exhaustion [40]. Thus, the signature of a poor response to immunotherapy is indicated by a state of early activation and should be thoroughly investigated in order to attain the desirable therapeutic response rates.

Finally, the limitations of this study are represented by the small number of cases and the absence of complete histopathological parameters of the melanoma-diagnosed relatives of the included patients. Moreover, the numerical reduction in the cases evaluated via QuPath, caused by preanalytical and analytical processing artifacts, may have interfered with the precision of the statistical analysis. However, Globocan states that approximately 1547 new cases of melanoma are diagnosed annually in Romania [41]; 0.2 to 8.6% of them are represented by multiple tumors [42], and 1–8% of all patients diagnosed with melanoma have at least a first-degree relative affected by the same oncological disease [43]. Corroborating the statistical data with the mention that our analysis only focused on the Romanian Dobruja region, the correlation between a high CD8-positive T cells percentage and poorer prognosis in familial and/or primary multiple melanomas remains valid. Moreover, a standardized method for the evaluation of CD8 positivity in intratumoral, as well as peritumoral, lymphocytic cellular populations should be proposed in order to minimize the difference between the visual abilities of the human eye and those of the software.

## 5. Conclusions

In the era of rapidly developing digital pathology, the use of automated image-scanning software is gaining popularity in the detriment of the human eye, because of the higher accuracy and replicability of the results obtained by the aid of the former technique. The present study was centered on Romanian individuals with multiple primary and/or familial melanoma and the main comparative findings between the human-eye assessments performed by experimental pathologists and the evaluations generated by the AI-mediated software exhibited notable variance, with considerable variation within each variable (“QuPath” and “Human”), and therefore did not harbor statistical significance. Nevertheless, the levels of accuracy of the evaluations provided by Qu-Path show promising results and should be explored further. A higher percentage of CD8-positive intratumoral lymphocytes versus stromal lymphocytes was positively associated with more metastatic sites. In conclusion, the CD8-lymphocytic phenotype possesses major significance in the realm of familial and multiple primary melanoma and may comprise a cost-effective investigation to help in the establishment of melanoma prognosis and the response to immunotherapy.

## Figures and Tables

**Table 1:** Immunohistochemical and molecular biology panel.

Immunohistochemical Antibody	Clone	Manufacturer	Dilution	Host, Clonality
CD8	SP16	Master Diagnostica	Ready-to-use (RTU) 7 mL	Rabbit, monoclonal
**FISH Probe**	**Clone**	**Manufacturer**	**Dilution**	**Additional Materials**
SPEC CDKN2A/CEN 9 Dual Color Probe	ZytoLight^®^	ZytoVision	RTU 0.2 mL	ZytoLight FISH Implementation Kit

**Table 2:** Demographic and clinical characteristics of the included participants (*n* = 23).

Demographic/Clinical Parameter	Percentual Values (Number of Patients)
Gender	
Male	47.82% (*n* = 11)
Female	52.17% (*n* = 12)
Age	
*Mean age*	62.30 ± 14.77
31–40	13.04% (*n* = 3)
41–50	17.39% (*n* = 4)
51–60	8.69% (*n* = 2)
61–70	21.73% (*n* = 5)
71–80	30.43% (*n* = 7)
81–90	8.69% (*n* = 2)
Area of residence	
Urban	60.86% (*n* = 14)
Rural	39.13% (*n* = 9)
Anatomic site of primary melanoma	
Head and neck	34.78% (*n* = 8)
Upper extremities	21.73% (*n* = 5)
Lower extremities	4.34% (*n* = 1)
Anterior/posterior thorax	39.13% (*n* = 9)

**Table 3:** Histopathological characterization of the evaluated melanoma lesions (*n* = 23).

Histopathological Parameter	Percentual Values (Number of Patients)
Histopathological melanoma subtype	
Superficial spreading melanoma	56.52% (*n* = 13)
Nodular melanoma	39.13% (*n* = 9)
Acral lentiginous melanoma	4.34% (*n* = 1)
Breslow depth (mm)	
Mean Breslow depth	4.88 ± 3.92
≤1.00	21.73% (*n* = 5)
1.01–2.00	13.04% (*n* = 3)
2.01–4.00	17.39% (*n* = 4)
≥4.01	47.82% (*n* = 11)
Ulceration	
Present	56.52% (*n* = 13)
Absent	43.47% (*n* = 10)
Mitotic rate (per mm^2^)	
Mean mitotic rate	3.04 ± 3.55
0	17.39% (*n* = 4)
1.00–1.99	34.78% (*n* = 8)
2.00–4.99	13.04% (*n* = 3)
≥5.00	34.78% (*n* = 8)
Lymphovascular invasion	
Present	56.52% (*n* = 13)
Absent	43.47% (*n* = 10)
Perineural invasion	
Present	21.73% (*n* = 5)
Absent	78.26% (*n* = 18)
Regression	
Present	17.39% (*n* = 4)
Absent	82.61% (*n* = 19)

**Table 4:** The inter-item covariance matrix between the QuPath and human-eye evaluation of CD8-positive TILs.

	QuPath	Human
QuPath	681,127	99,236
Human	99,236	803,526

**Table 5:** Intraclass correlation coefficient.

	Intraclass Correlation ^b^	95% Confidence Interval		F Test with True Value 0			
		Lower Bound	Upper Bound	Value	df1	df2	Sig
Single Measures	0.134 ^a^	−0.429		0.622	1.309	12	12
Average Measures	0.236 ^c^	−1.504		0.767	1.309	12	12

^a^ The estimator is the same, whether the interaction effect is present or not. ^b^ Type C intraclass correlation coefficients using a consistency definition. The between-measure variance is excluded from the denominator variance. ^c^ This estimate is computed assuming the interaction effect is absent, because it is not estimable otherwise.

## Data Availability

The data generated in the present study are included in the figures and/or tables of this article.
